# Clinical Characteristics of Elderly People with Osteoporotic Vertebral Compression Fracture Based on a 12-Year Single-Center Experience in Korea

**DOI:** 10.3390/geriatrics7060123

**Published:** 2022-10-31

**Authors:** Seung-Kwan Lee, Deuk-Soo Jun, Dong-Keun Lee, Jong-Min Baik

**Affiliations:** 1 Department of Orthopedic Surgery, Division of Spine, Incheon Sarang Hospital, Incheon 22135, Korea; 2 Department of Orthopedic Surgery, Division of Spine, Gachon University Gil Medical Center, Incheon 21565, Korea

**Keywords:** osteoporosis, osteoporotic fracture, vertebral compression fracture, bone mineral density, dual-energy X-ray absorptiometry

## Abstract

In an aging human population, osteoporotic vertebral compression fracture (OVCF) frequently occurs. We conducted this retrospective study to analyze the clinical characteristics of elderly people with OVCF who underwent percutaneous vertebroplasty or kyphoplasty over a 12-year period at a single medical center in Korea. Between 2007 and 2019, A total of 868 patients (*n* = 868) were treated at our institution. We assessed 600 of these patients as eligible for study purposes and divided them into three groups: Group A (spine and hip T-scores ≤−2.5; *n* = 332); Group B (spine T-scores ≤−2.5; *n* = 189); and Group C (hip T-scores ≤−2.5; *n* = 79). The baseline characteristics of the patients included age, sex, body mass index (BMI), past history of steroid use, alcohol consumption, use of osteoporosis therapy, smoking, and treatment for OVCF. We compared these characteristics between the three groups. We found that the mean patient age was significantly higher in Group A, compared with Group B, and significantly lower in Group B, compared with Group C. We also found significant differences in the male-to-female ratio and mean body mass index between the three groups. In conclusion, we suggest that special attention should be paid to factors closely associated with spine and hip T-scores when evaluating elderly people with OVCF and determining appropriate treatment.

## 1. Introduction

Both men and women are vulnerable to bone loss with aging because of age-related factors such as menopause in women, which can lead to the occurrence of osteoporosis [1]. Osteoporosis is defined as low bone mass with structural loss of systemic skeletal tissue and is further characterized by deterioration of microarchitecture, followed by increased bone fragility and consequent susceptibility to fracture [1,2]. Osteoporotic fracture (OF) is one of the most serious medical issues affecting elderly people; it is characterized by high morbidity, and seriously impairs patient quality of life [3]. The incidence of OF rises with increased life expectancy. In addition, vertebral fracture is one of the most common types of OF [4].

Osteoporotic vertebral compression fracture (OVCF) is a common and severe consequence of osteoporosis [5,6]. It is a type of frailty fracture that affects individuals aged ≥50 years with an estimated prevalence of 30–50% [7]. A total of 25% of women aged ≥65 years and 40% of those aged ≥80 years present with symptoms that are suggestive of OVCF [8]. A diagnosis of OVCF is frequently missed because it also affects individuals with no history of trauma [9]. Patients with OVCF are vulnerable to severe pain, disability, and increased mortality; they are also at a four-times-greater risk of secondary fracture [10,11,12]. Percutaneous vertebroplasty (VP) or kyphoplasty (KP) procedures are routinely performed to treat patients with OVCF. However, approximately 20% of patients who do undergo surgery experience a recurrence of vertebral compression fracture (VCF) within one year of the first fracture [13]. This is a serious health problem that represents a major social and economic burden worldwide [14,15]. Previous studies have shown that risk factors of OVCF include the following: an age of ≥50 years; being female and elderly; black ethnicity; alcohol consumption; being a current smoker; a past history of taking drugs (e.g., glucocorticoids and anti-thyroid or anti-tubercular medications); a past history of fractures; short stature; low body mass index (BMI); rheumatoid arthritis; vitamin D deficiency; and a family history of VCF in the first degree of relatives [16,17,18,19].

To date, we have treated elderly OVCF patients with percutaneous VP or KP procedures [20,21,22,23,24,25]. However, in the diagnosis and management of OVCF patients, there is commonly a discordance between the T-scores of the spine and hip. Many studies have analyzed the prevalence of this T-score discordance and associated factors in terms of the management of osteoporosis. By contrast, fewer studies have considered clinical factors affecting T-score discordance in OVCF patients. We, therefore, carried out this retrospective study of elderly OVCF patients treated at our institution over a 12-year period, to assess clinical characteristics with respect to T-score discordance.

## 2. Materials and Methods

### 2.1. Study Patients and Setting

A total of 868 patients (*n* = 868) were treated by percutaneous VP or KP at our medical institution during a 12-year period between 2007 and 2019. For the purposes of the present study, we included patients who satisfied the following inclusion criteria:

(1) Korean adults aged ≥50 years.
(2) Patients with T-scores of ≤−2.5 on dual-energy X-ray absorptiometry (DEXA).
(3) Patients with a confirmed diagnosis of OVCF.
(4) Patients with available medical records.

We also applied exclusion criteria, as follows:

(1) Patients with T-scores of >−2.5 on dual-energy X-ray absorptiometry (DEXA).
(2) Patients with lumbar fusion, multi-segmental vertebral fractures, or bilateral femoral fractures.
(3) Patients undergoing hip arthroplasty.
(4) Patients lost to follow-up.
(5) Patients with missing data.

In all, we included a total of 600 eligible patients (*n* = 600). Our study was approved by the Institutional Review Board of our medical institution (GAIRB No. 2021-358) and conducted in compliance with the relevant ethics guidelines. Written informed consent was waived due to its retrospective nature. All the procedures described herein were performed in accordance with the 1964 Declaration of Helsinki and its later amendments or comparable ethical standards.

### 2.2. Diagnostic Work-Up for OVCF in the Elderly

Currently, the measurement of bone mineral density (BMD) at the lumbar spine and hip is the gold-standard method for making a diagnosis of osteoporosis. However, total BMD T-scores can identify <50% of patients who will develop an OF even if they are diagnosed with osteoporosis [26]. We, therefore, used plain radiography and computed tomography (CT) to make an initial diagnosis of OVCF. A CT scan offers a convenient diagnostic modality to identify the presence of a fracture. However, it is difficult to make a differential diagnosis between old and new fractures. For this reason, we have routinely carried out magnetic resonance imaging (MRI) in addition to a bone scan to diagnose OVCF in elderly individuals with vertebral diseases but with no past history of trauma [20,21,22,23,24,25,27].

### 2.3. DEXA Protocol for the Measurement of the BMD of the Lumbar Spine and Hip

DEXA is currently the most popular modality in the diagnosis of osteoporosis, allowing fast, accurate and non-invasive measurement of BMD [28]. In the current study, we measured BMD using the Prodigy DXA scanner (GE Healthcare, Madison, WI, USA) and used the manufacturer’s software for analysis purposes. To this end, we scanned vertebrae L1–L4 and the left hip using posteroanterior projections when patients were placed in the supine position. We also determined the femoral neck and total hip to be regions of interest in measuring hip BMD. We based our T-score reference ranges on those of a previous study [29].

### 2.4. Patient Evaluation and Criteria

**Figure 1:**
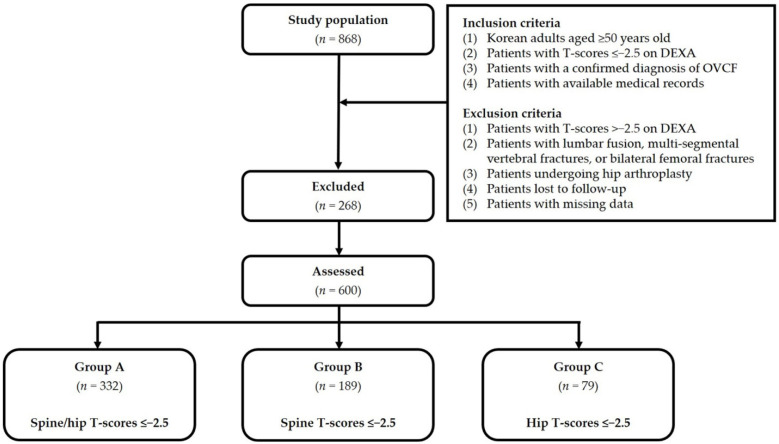
Disposition of the study patients. Abbreviations: OVCF—osteoporotic vertebral compression fracture; DEXA—dual-energy X-ray absorptiometry.

For our analysis of discordance in BMD between hip and spine, we divided our study patients into three groups, as follows: Group A (spine and hip T-scores ≤−2.5; *n* = 332); Group B (spine T-scores ≤−2.5; *n* = 189); and Group C (hip T-scores ≤−2.5; *n* = 79). Figure 1 shows the disposition of the analyzed patients. Baseline characteristics include age, sex, BMI, a past history of steroid use, use of osteoporosis therapy, smoking, alcohol consumption, and treatment for OVCF. All characteristics were obtained from a retrospective review of patient medical records.

### 2.5. Statistical Analysis of the Patients’ Data

We expressed all data as mean ± SD or the number of the patients with percentage, where appropriate. We analyzed continuous variables using a one-way analysis of variance (ANOVA). We also analyzed categorical variables using the χ^2^- or Fisher’s exact test. We carried out all statistical analyses using the SPSS Ver. 18 for Windows (SPSS, Inc., Chicago, IL, USA). We considered a *p*-value of <0.05 to be statistically significant.

## 3. Results

**Figure 2:**
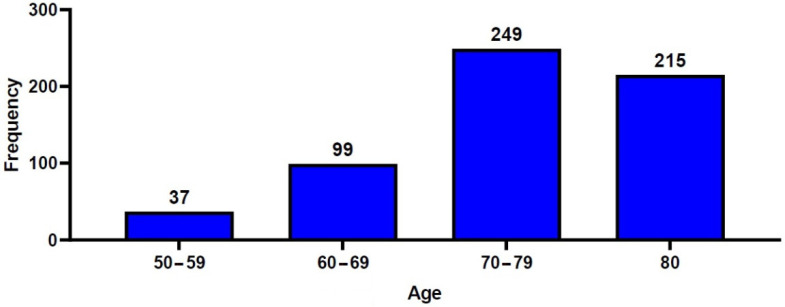
Distribution of the patients’ age.

A total of 600 patients with OVCF were included in the current retrospective analysis, consisting of 111 men (18.5%) and 489 women (81.5%), with a mean age of 75.6 ± 9.1 years. The study population consisted of 37 patients (6.2%) aged between 50 and 59 years, 99 (16.5%) aged between 60 and 69 years, 249 (41.5%) aged between 70 and 79 years, and 215 (35.8%) aged ≥80 years (Figure 2). A total of 261 patients (43.5%) underwent percutaneous VP, and 339 patients (56.5%) underwent percutaneous KP. Table 1 and Table 2 show the baseline characteristics of the study patients.

**Figure 3:**
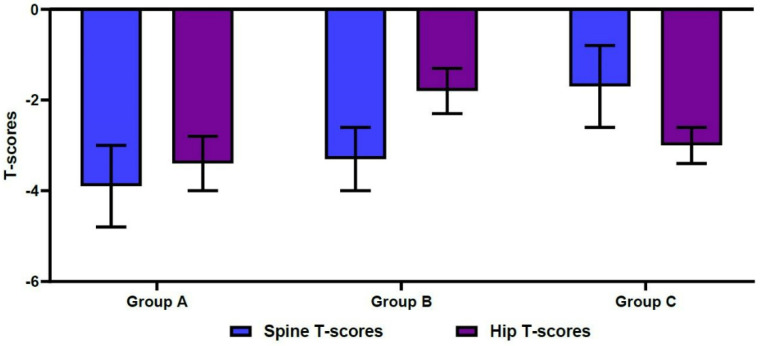
Differences in spine/hip T-scores on dual-energy X-ray absorptiometry.

**Figure 4:**
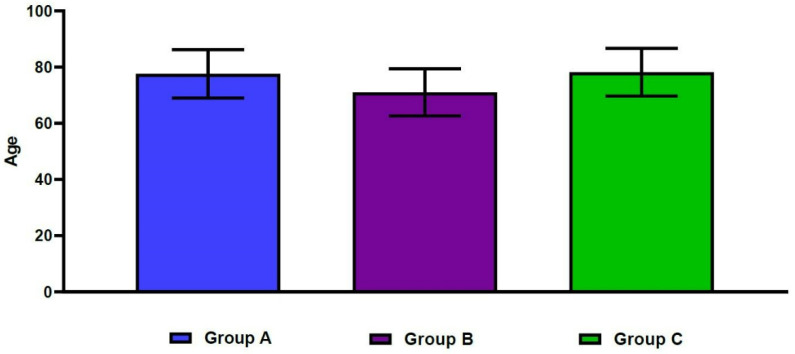
Differences in age between the three groups.

The mean spine T-scores were −3.9 ± 0.9 in Group A, −3.3 ± 0.7 in Group B, and −1.7 ± 0.9 in Group C. For the same groups, mean hip T-scores were −3.4 ± 0.6, −1.8 ± 0.5, and −3.0 ± 0.4, respectively (Figure 3). These differences were statistically significant (*p* < 0.001). Table 2 shows significant differences in age, sex, and BMI between the three groups (*p* < 0.05). In more detail, the mean age was significantly higher in Group A, compared with Group B (77.6 ± 8.6 vs. 71.0 ± 8.4 years old, respectively; *p* < 0.001). The mean age was also significantly lower in Group B, compared with Group C (71.0 ± 8.4 vs. 78.2 ± 8.5 years old, respectively; *p* < 0.001) (Figure 4). We also found significant differences in the male-to-female ratios between the three groups (*p* < 0.001). Finally, the mean BMI was significantly lower in Group A, compared with both Group B and Group C (21.1 ± 3.4 vs. 22.9 ± 3.1 and 22.7 ± 2.8 kg/m^2^, respectively; *p* < 0.001).

## 4. Discussion

Today, the rapid progression of aging is a worldwide phenomenon. Korea is expected to become a “super-aged” society in 2026. Therefore, there is a growing interest in aging-related diseases. Of these, osteoporosis deserves special attention. In 2017, the Korean Society for Bone and Mineral Research published the “Osteoporosis and Osteoporotic Fracture Fact Sheet in Korea”, which revealed that osteoporosis occurred in 37% of Korean women aged 50 years or more, and in 7.5% of Korean men aged 50 years or more. The document also reported a 4% increase in the incidence of OF every year between 2008 and 2013 and predicted that the incidence of osteoporosis would rise by a factor of two amongst women with every 10-year increase in age so that 68.5% of women aged 70 years or more would eventually develop osteoporosis [30].

BMD is measured by means of DEXA, which is used to make a diagnosis of osteoporosis and to assess the risk of fracture [31,32]. The International Society for Clinical Densitometry (ISCD) recommends the measurement of BMD to make a diagnosis of osteoporosis at the hip and lumbar spine [33]. In a clinical setting, however, raw BMD values (g/cm^2^) are not used when assessing the status of bony structures and predicting the risk of fracture. Instead, they are expressed as the number of standard deviations above or below the normal values obtained from younger individuals—aged between 20 and 39 years—from the same ethnic population. By such means, T-scores can be calculated [34]. T-score is defined as the standard deviation (SD) score of the observed BMD in comparison with that of a normal young adult [35]. Previous studies have incorporated DEXA-derived cut-off values of T-scores ranging from −2.5 to −4.0 standard deviation (SD) measured from the spine and hip [36,37]. The World Health Organization has defined osteoporosis based on DEXA measurement; according to the WHO, osteoporosis is defined based on T-scores of ≤−2.5 SD [38]. The ISCD has recommended that a diagnosis of osteoporosis should be based on the lowest T-scores measured at the spine, total hip, and femoral neck [39]. However, there is often discordance in the measurement of T-scores; specifically, the presence of different categories of T-scores between the hip and spine. Several studies have sought to identify clinical factors that may predict such discordance in the elderly population [32]. To date, only a few studies have sought to identify clinical factors associated with T-score discordance in OVCF patients.

Previous studies have suggested that useful indicators of BMD changes in elderly people include aging itself, postmenopausal status, metabolic and endocrine diseases, inadequate physical activity, BMI, smoking, vitamin D deficiency, thyroid function, serum ferritin levels and a family history of osteoporosis [40,41,42]. These findings are in line with our results; we found significant differences in age, sex, and BMI between our three groups.

In our study populations, we found a significantly higher association of mean age with a combined spine T-score of ≤−2.5 and hip T-score of ≤−2.5, compared with a spine T-score of ≤−2.5 only (*p* < 0.001). We also found a significantly lower association of mean age with a spine T-score of ≤−2.5 alone, but not with a hip T-score of ≤−2.5 alone (*p* < 0.001). These results indicate that age is more closely associated with a hip T-score of ≤−2.5, compared with a spine T-score of ≤−2.5. Osteoporosis can often be accompanied by osteoarthritis in elderly women [43]. Moreover, aging-related degenerative changes in the spine, such as vertebral osteophytes, vertebral endplate sclerosis, facet joint sclerosis, osteochondrosis, and aortic calcification, can elevate spine T-scores above their actual values [44]. Such degenerative changes may cause abnormal calcium deposition within the field of the DEXA region of interest [45]. The authors of [46] reported that the BMD of fractured vertebrae was significantly higher than that of non-fractured vertebrae. The present study involved a cohort of patients with OVCF; our findings suggest that hip T-score might be a more reliable indicator of osteoporosis compared with spine T-score in more elderly population groups.

We found a significant difference in the male-to-female ratio between the three groups (*p* < 0.001). This was highest in association with a combined spine T-score of ≤−2.5 and hip T-score of ≤−2.5, and lowest in association with a spine T-score of ≤−2.5 alone. These results indicate that the female sex is more closely associated with a spine T-score of ≤−2.5 than a hip T-score of ≤−2.5.

As societies gradually transform into aging societies, the age of physical activity in men is increasingly prolonged [47]. In this regard, the lower age of the male patients in our Group B, compared with the other groups, may offer a biomechanical explanation of increased hip BMD values and discordance with values for the spine. In biomechanical terms, physical activity increases bone mass by means of a physiological stimulus caused by stress transfer [48]. The hip and femur are body parts that bear weight more directly than the lumbar spine. It is possible to maintain bone mass due to stress transfer to the hip and femur directly in weight-bearing, and this can produce a relatively higher T-score at the hip, compared with the spine. This is a matter of interest when considering age factors and male-to-female ratios in an aging society. With the development of modern medicine, the effects of age might be increased.

Our results also showed that mean BMI was significantly lower in association with a spine T-score of ≤−2.5 and a hip T-score ≤−2.5 combined, compared with a spine T-score of ≤−2.5 alone, or a hip T-score of ≤−2.5 alone. However, the measured mean BMI of each group was within the normal range, so our results do not definitively determine any association with BMI. However, our findings are in agreement with those of a previous study that suggested that low BMI might serve as a risk factor for low BMD [49].

Our results cannot be generalized; the limitations of the current study are as follows: First, our study was retrospective in design. This made additional analysis regarding comorbidity and inter-relationships with other osteoporotic fractures difficult. The impacts of comorbidity and other osteoporotic fractures should be considered in future studies. In addition, propensity score-matching analysis might be conducted to strengthen study results by removing covariate imbalance. Second, the number of patients in Group C (*n* = 79) was much smaller, compared with our Group A (*n* = 332) or Group B (*n* = 189). For this reason, we cannot rule out the possibility of comparison bias. Third, our medical institution is a tertiary-care, multi-specialty, 1600-bed hospital located in a metropolitan area in Korea. Again, this raises the possibility of selection bias. Nevertheless, our results are of importance; to our knowledge, this is the first large-scale study to describe the clinical characteristics regarding BMD discordance in elderly people with OVCF in Korea.

## 5. Conclusions

Our results indicate that special attention should be paid to factors that are closely associated with spine/hip T-scores for the appropriate evaluation and management of the elderly with OVCF. In diagnosing osteoporosis for OVCF patients, the hip T-score is a more reliable indicator of osteoporosis in more elderly patients. Furthermore, these results may be helpful in determining the direction of treatment and management of elderly patients with OVCF.

## Figures and Tables

**Table 1:** Baseline characteristics of the patients (*n* = 600).

Variables	Values
Age (years old)	75.6 ± 9.1
50–59	37 (6.2%)
60–69	99 (16.5%)
70–79	249 (41.5%)
≥80	215 (35.8%)
Sex	
Men	111 (18.5%)
Women	489 (81.5%)
* Postmenopausal women	475 (97.1%)
BMI (kg/m^2^)	21.9 ± 3.4
Smoking	
Yes	42 (7.0%)
No	558 (93.0%)
Alcohol consumption	
Yes	59 (9.8%)
No	541 (90.2%)
Steroid therapy	
Yes	40 (6.7%)
No	560 (93.3%)
Osteoporosis therapy	
Yes	584 (84.0%)
No	96 (16.0%)
DEXA T-scores	
Spine	−3.4 ± 1.1
Hip	−2.8 ± 0.9
OVCF treatment	
VP	261 (43.5%)
KP	339 (56.5%)

Abbreviations: BMI—body mass index; DEXA—dual-energy X-ray absorptiometry; OVCF—osteoporotic vertebral compression fracture; VP—vertebroplasty; KP—kyphoplasty. Values are mean ± standard deviation or the number of patients with percentage, where appropriate. * Percentile of postmenopausal women.

**Table 2:** Differences in baseline characteristics of the patients between the three groups (*n* = 600).

Variables	Values			*p*-Value
	Group A (*n* = 332)	Group B (*n* = 189)	Group C (*n* = 79)	
Age (years)	77.6 ± 8.6	71.0 ± 8.4	78.2 ± 8.5	<0.001 *
50–59	12 (3.6%)	23 (12.2%)	2 (2.5%)	<0.001 *
60–69	41 (12.3%)	49 (25.9%)	9 (11.4%)	
70–79	130 (39.2%)	88 (46.6%)	31 (39.2%)	
≥80	149 (44.9%)	29 (15.3%)	37 (46.8%)	
Sex				
Male-to-female ratio	0.14	0.39	0.25	<0.001 *
Men	42 (12.7%)	53 (28.0%)	16 (20.3%)	
Women	290 (87.3%)	136 (72.0%)	63 (79.7%)	
Postmenopausal women	283 (85.2%)	131 (69.3%)	61 (77.2%)	0.001 *
BMI (kg/m^2^)	21.1 ± 3.4	22.9 ± 3.1	22.7 ± 2.8	<0.001 *
Smoking				
Yes	17 (5.1%)	20 (10.6%)	5 (6.3%)	0.114
No	315 (94.9%)	169 (89.4%)	74 (93.7%)	
Alcohol consumption				
Yes	31 (9.3%)	22 (11.6%)	6 (7.6%)	0.539
No	301 (90.7%)	167 (88.4%)	73 (92.4%)	
Steroid therapy				
Yes	20 (6.0%)	15 (7.9%)	5 (6.3%)	0.696
No	312 (94.0%)	174 (92.1%)	74 (93.7%)	
Osteoporosis therapy				
Yes	277 (83.4%)	150 (84.7%)	67 (84.8%)	0.125
No	55 (16.6%)	29 (15.3%)	12 (15.2%)	
OVCF treatment				
VP	163 (49.1%)	94 (49.7%)	34 (43.0%)	0.575
KP	169 (50.9%)	95 (50.3%)	45 (57.0%)	

Abbreviations: BMI—body mass index; OVCF—osteoporotic vertebral compression fracture; VP—vertebroplasty; KP—kyphoplasty. Values are mean ± standard deviation or the number of patients with percentage, where appropriate. * Statistical significance at *p* < 0.05.

## Data Availability

The data presented in this study are available upon reasonable request from the corresponding author. The data are not publicly available because of privacy concerns.
